# Supplementary material for: Host Immune Response Driving SARS-CoV-2 Evolution
Source: Viruses. 2020 Sep 27;12(10):1095. doi: 10.3390/v12101095 (PMC7599751; doi:10.3390/v12101095)
Supplement: Supplementary file 1 [file viruses-12-01095-s001.zip › 2019-nCoV-GeneEditing_Supporting_5.pdf]

Supporting Information
Host immune response driving SARS-CoV-2 evolution

Rui Wang[1], Yuta Hozumi[1], Yong-Hui Zheng[2], Changchuan Yin[3] *, and Guo-Wei Wei[1,4,5] †

[1] Department of Mathematics, Michigan State University, MI 48824, USA

[2] Department of Microbiology and Molecular Genetics, Michigan State University, MI 48824, USA

[3] Department of Mathematics, Statistics, and Computer Science, University of Illinois at Chicago, Chicago, IL 60607, USA

[4] Department of Biochemistry and Molecular Biology Michigan State University, MI 48824, USA

[5] Department of Electrical and Computer Engineering Michigan State University, MI 48824, USA

September 4, 2020

Contents

S1 Supplementary Figures **1**

S1.1 The distribution of 12 SNP types among non-unique mutations 1

S1.2 The distribution of 12 SNP types between each pair of 10 coronaviruses 1

S1.3 SNP frequencies on 4-mer motifs 2

S2 Supplementary Tables **18**

*Address correspondences to Changchuan Yin. E-mail:cyin1@uic.edu

†Address correspondences to Guo-Wei Wei. E-mail:wei@math.msu.edu

S1 Supplementary Figures

S1.1 The distribution of 12 SNP types among non-unique mutations

In this section, we illustrate the distributions of 12 SNP types of SARS-CoV-2 among non-unique mutations in different age groups, gender groups, and geographic locations from Figure S1 to Figure S9.

Figure S1: The distribution of 12 SNP types of SARS-CoV-2 among non-unique mutations in different age groups globally.

Figure S2: The distribution of 12 SNP types of SARS-CoV-2 among non-unique mutations in different gender groups globally.

Figure S3: The distribution of 12 SNP types of SARS-CoV-2 among non-unique mutations in different age groups among female patients globally.

Figure S4: The distribution of 12 SNP types of SARS-CoV-2 among non-unique mutations in different age groups among male patients globally.

Figure S5: The distribution of 12 SNP types of SARS-CoV-2 among non-unique mutations in six continents.

Figure S6: The distribution of 12 SNP types of SARS-CoV-2 among non-unique mutations from the United Kingdom.

Figure S7: The distribution of 12 SNP types of SARS-CoV-2 among non-unique mutations from the United States.

Figure S8: The distribution of 12 SNP types of SARS-CoV-2 among non-unique mutations from the Australia.

Figure S9: The distribution of 12 SNP types of SARS-CoV-2 among non-unique mutations from the India. Figure S10: The distribution of 12 SNP types of SARS-CoV-2 among unique mutations from the United Kingdom.

Figure S11: The distribution of 12 SNP types of SARS-CoV-2 among unique mutations from the United States.

Figure S12: The distribution of 12 SNP types of SARS-CoV-2 among unique mutations from the Australia.

Figure S13: The distribution of 12 SNP types of SARS-CoV-2 among unique mutations from the India.

S1.2 The distribution of 12 SNP types between each pair of 10 coronaviruses

In this section, we analyze the SNP between each pair of the complete sequences of 10 coronaviruses: SARS-CoV (2003) [1], Bat-SL-BM48-31 (2008) [2], Bat-SL-RsSHC014 (2011) [3], Bat-SL-WIV1 (2012) [3], Bat-RaTG13(2013) [4], Bat-SL-As6526 (2014) [5], Bat-SL-CoVZXC21 (2015) [6], Bat-16BO133 (2016) (GeneBank), Bat-SL-CoVZC45 (2017) [6], SARS-CoV-2 (2019) [7]. The C>T over T>C ratio between each pair of 10 coronaviruses are illustrated in Figure S14. For example, the colored square located in the SARS-CoV row and SARS-CoV-2 column shows the C>T over T>C ratio of the SARS-CoV-2 genome with the SARS-CoV genome as a reference.

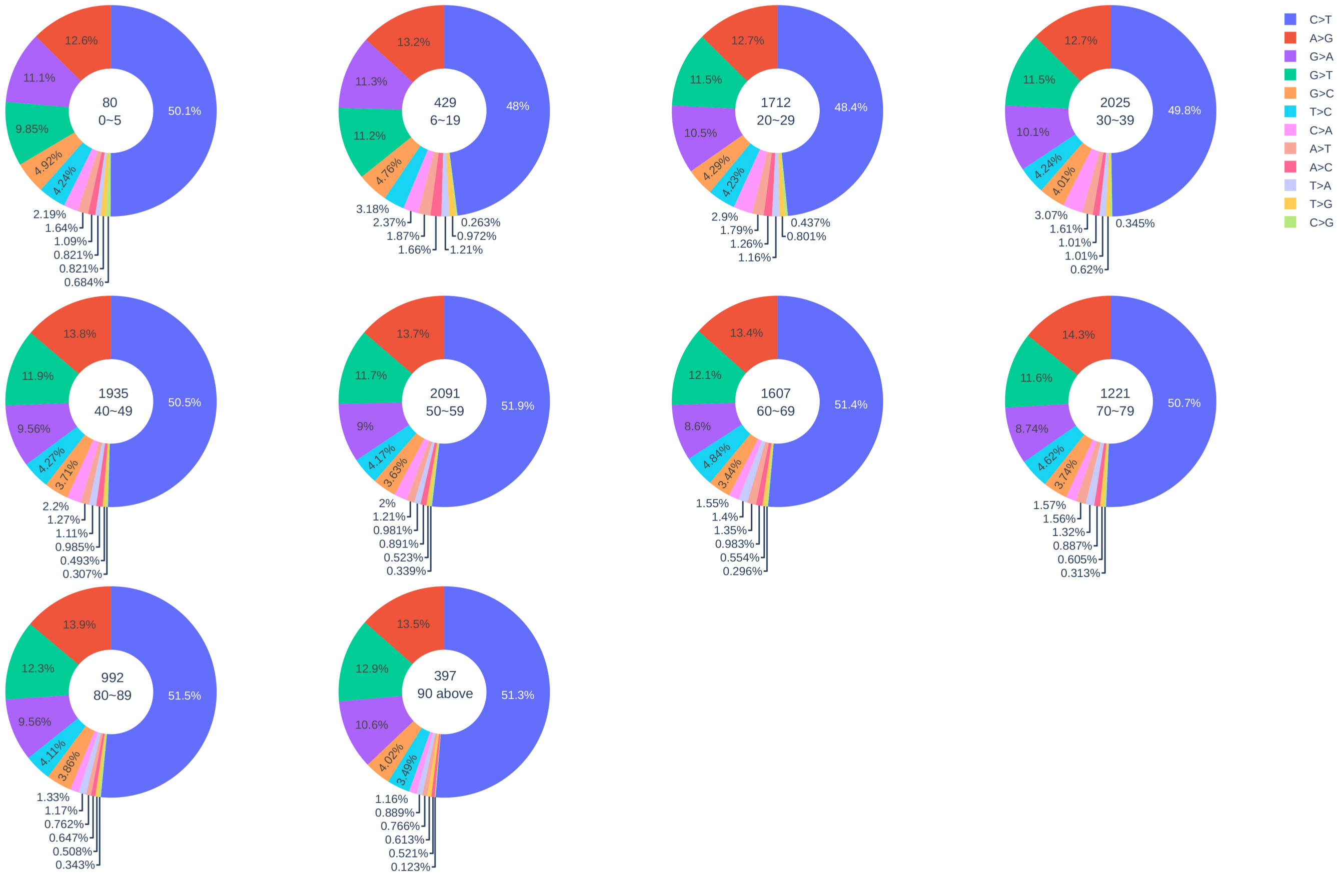

Figure S1: The distribution of 12 SNP types of SARS-CoV-2 among non-unique mutations in different age groups globally. The text inside each circle represents for the total number of records that have the age information in different age groups.

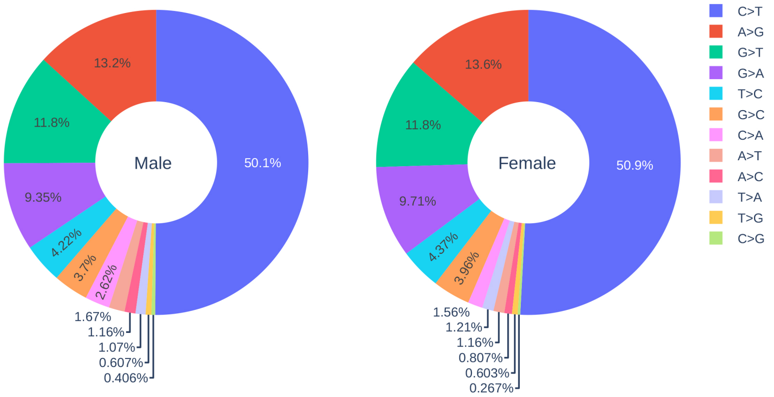

Figure S2: The distribution of 12 SNP types of SARS-CoV-2 among non-unique mutations in different gender groups globally. The text inside each circle represents for the total number of records that have the gender information in different gender groups.

S1.3 SNP frequencies on 4-mer motifs

In this section, we present 4-mer sequence contexts from Figure S15 to Figure S30.

Figure S15 to Figure S18: SNP frequencies at the first position (A, T, C, G) of 4-mer motifs.

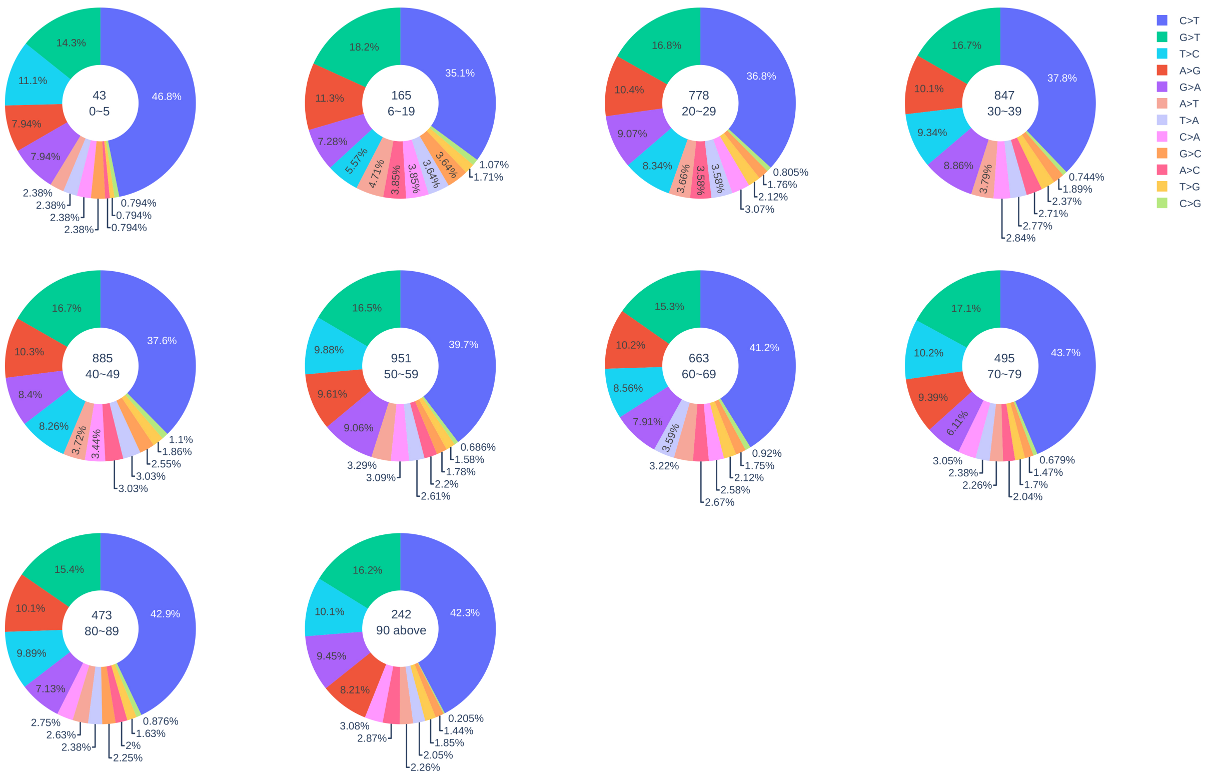

Figure S3: The distribution of 12 SNP types of SARS-CoV-2 among non-unique mutations in different age groups among female patients globally. The text inside each circle represents for the total number of records that have the age information in different age groups.

Figure S19 to Figure S22: SNP frequencies at the second position (A, T, C, G) of 4-mer motifs.

Figure S23 to Figure S26: SNP frequencies at the third position (A, T, C, G) of 4-mer motifs.

Figure S27 to Figure S30: SNP frequencies at the fourth position (A, T, C, G) of 4-mer motifs.

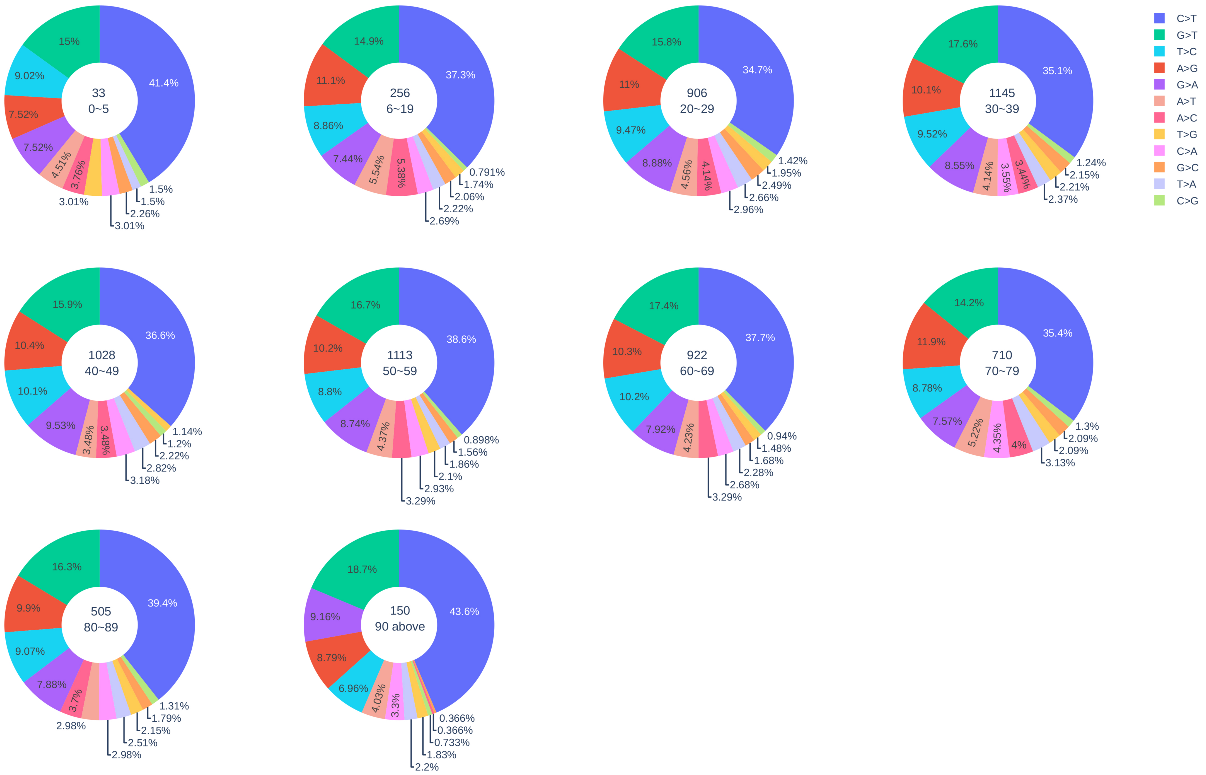

Figure S4: The distribution of 12 SNP types of SARS-CoV-2 among non-unique mutations in different age groups among male patients globally. The text inside each circle represents for the total number of records that have the age information in different age groups.

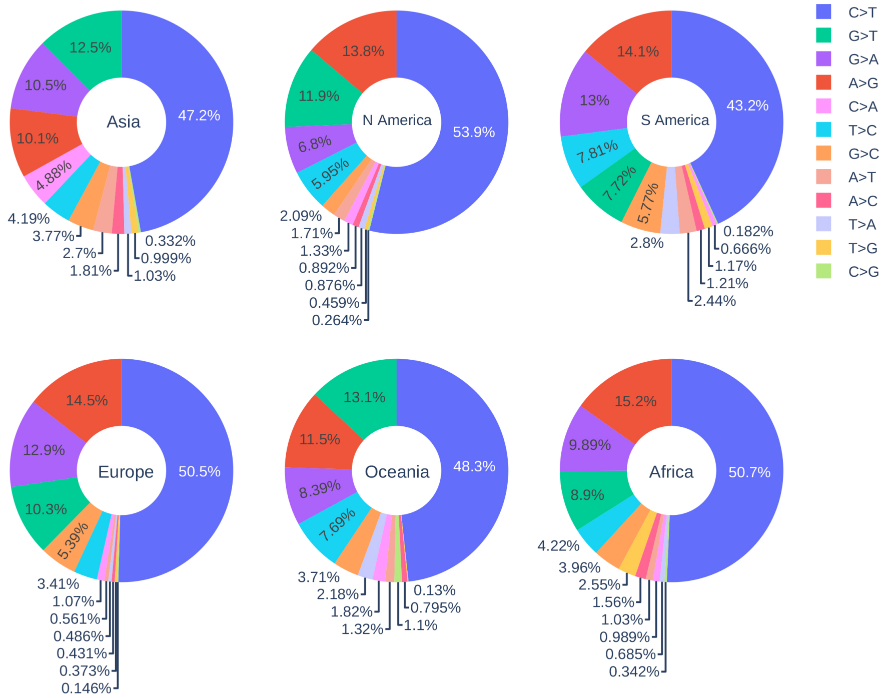

Figure S5: The distribution of 12 SNP types of SARS-CoV-2 among non-unique mutations in six continents. The text inside each circle represents for the total number of records in each continent.

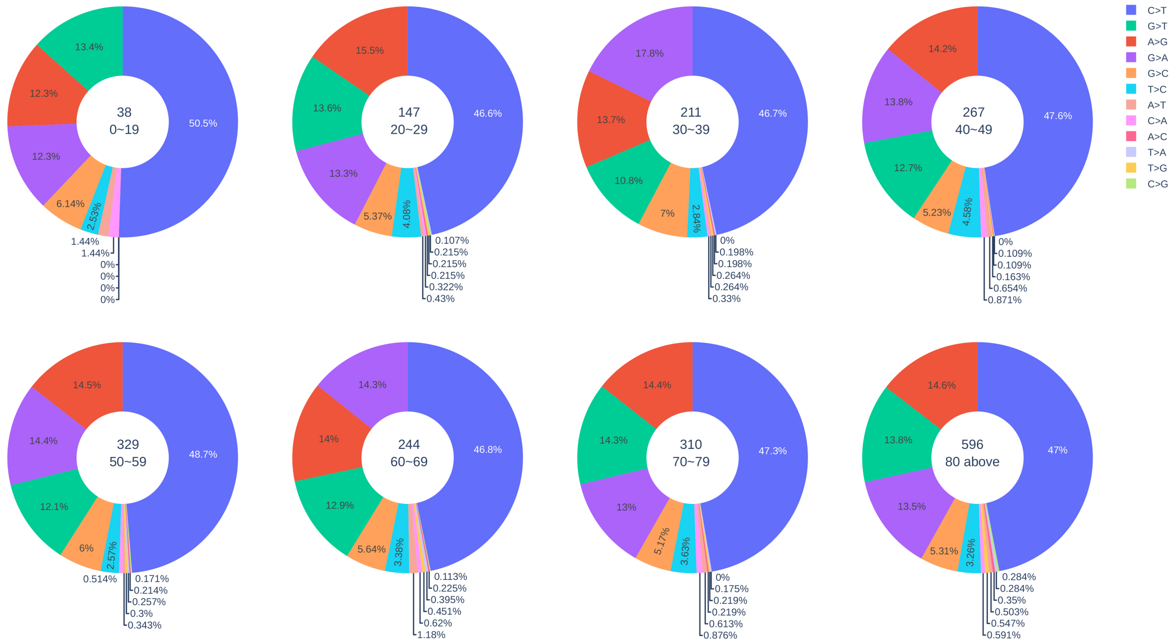

Figure S6: The distribution of 12 SNP types of SARS-CoV-2 among non-unique mutations from the United Kingdom. The text inside each circle represents for the total number of records in different age groups.

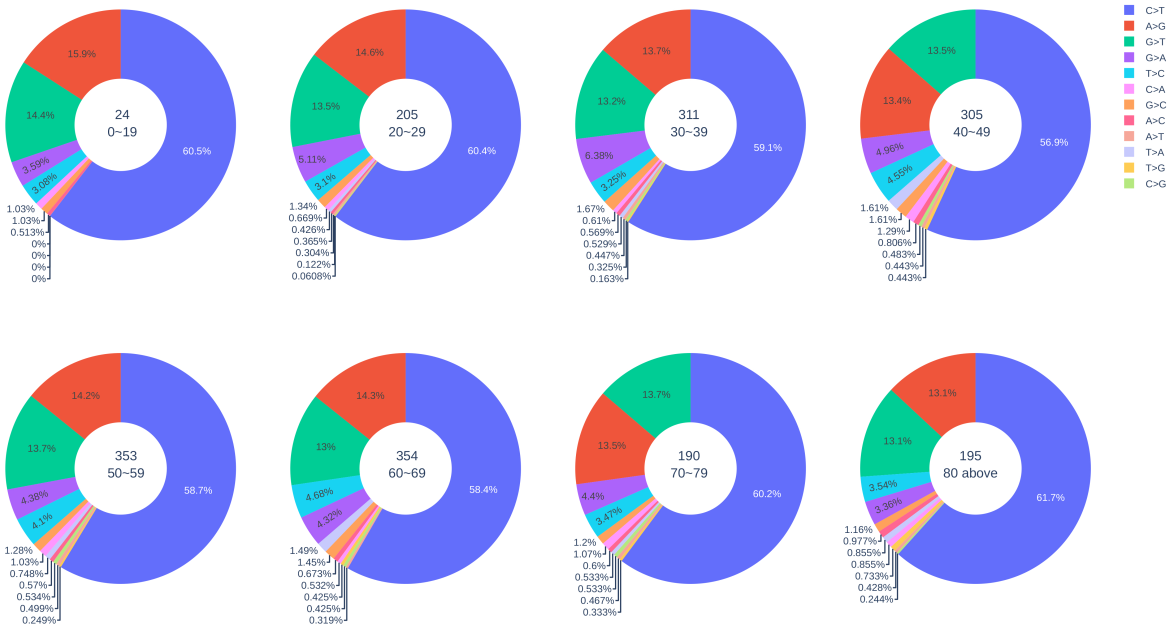

Figure S7: The distribution of 12 SNP types of SARS-CoV-2 among non-unique mutations from the United States. The text inside each circle represents for the total number of records in different age groups.

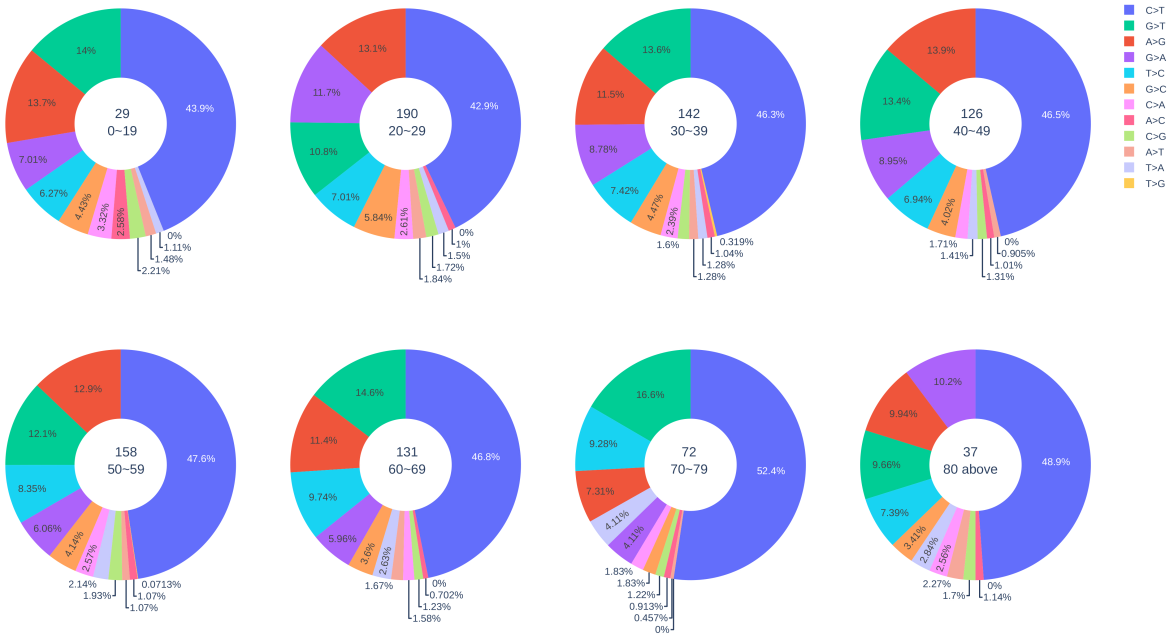

Figure S8: The distribution of 12 SNP types of SARS-CoV-2 among non-unique mutations from Australia. The text inside each circle represents for the total number of records in different age groups.

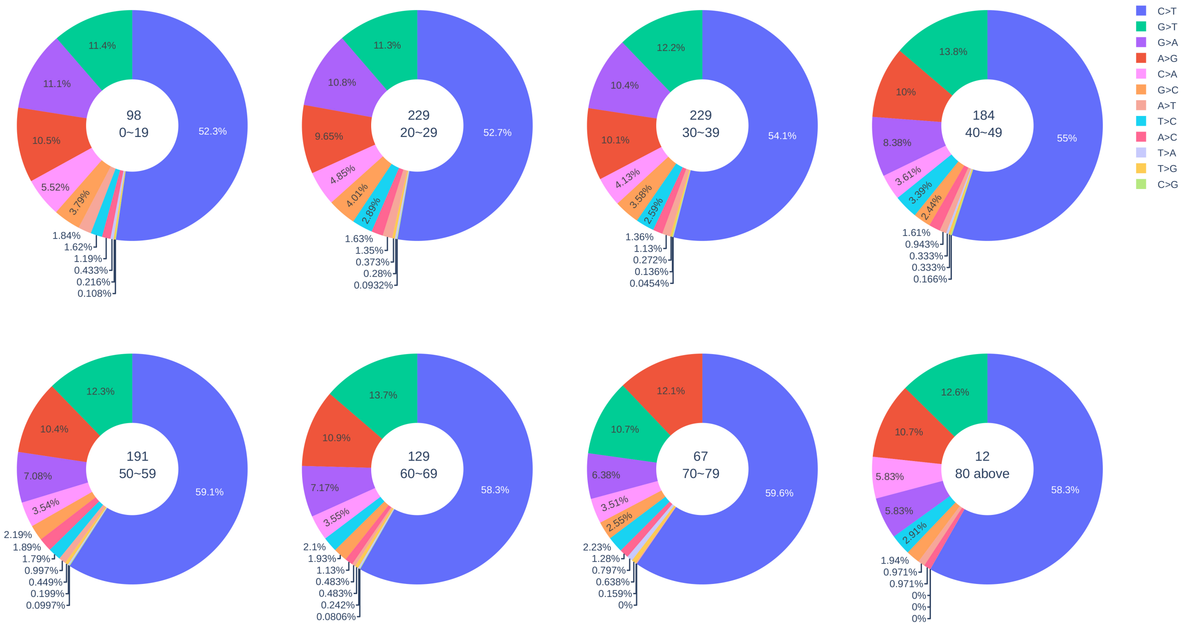

Figure S9: The distribution of 12 SNP types of SARS-CoV-2 among non-unique mutations from India. The text inside each circle represents for the total number of records in different age groups.

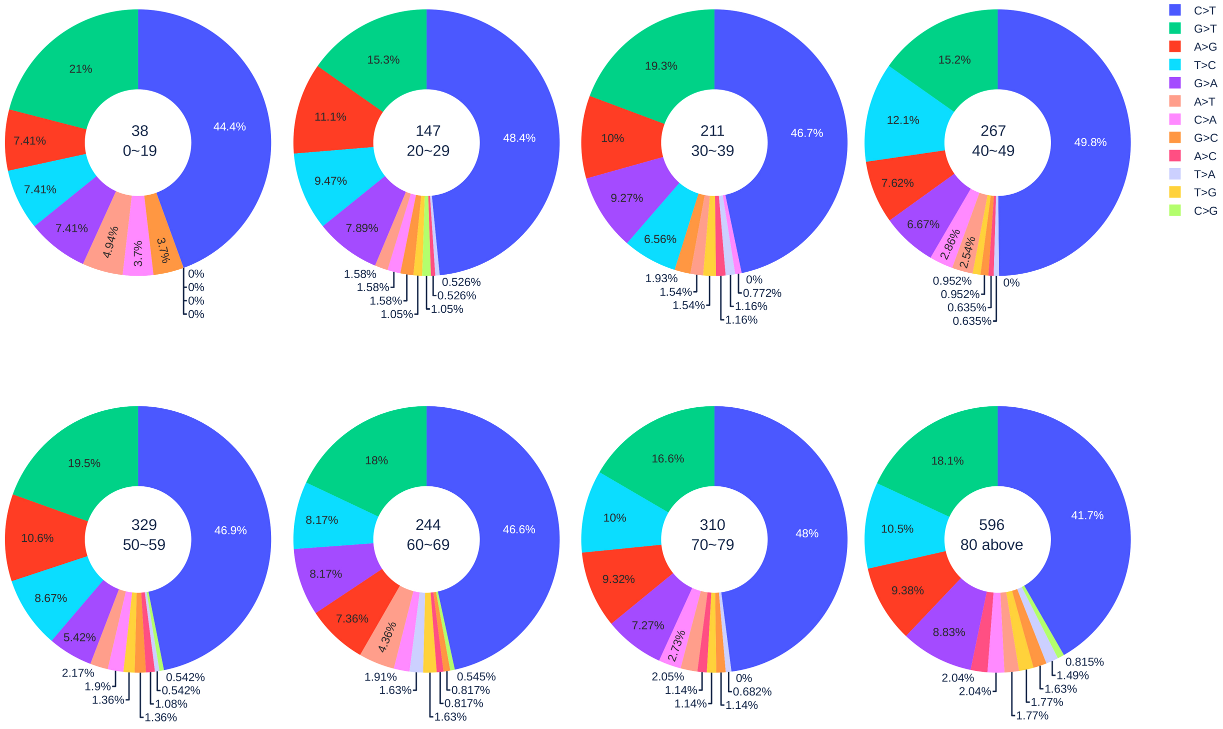

Figure S10: The distribution of 12 SNP types among unique mutations in the SARS-CoV-2 genome isolates from the United Kingdom. The text inside each circle represents for the total number of records in different age groups.

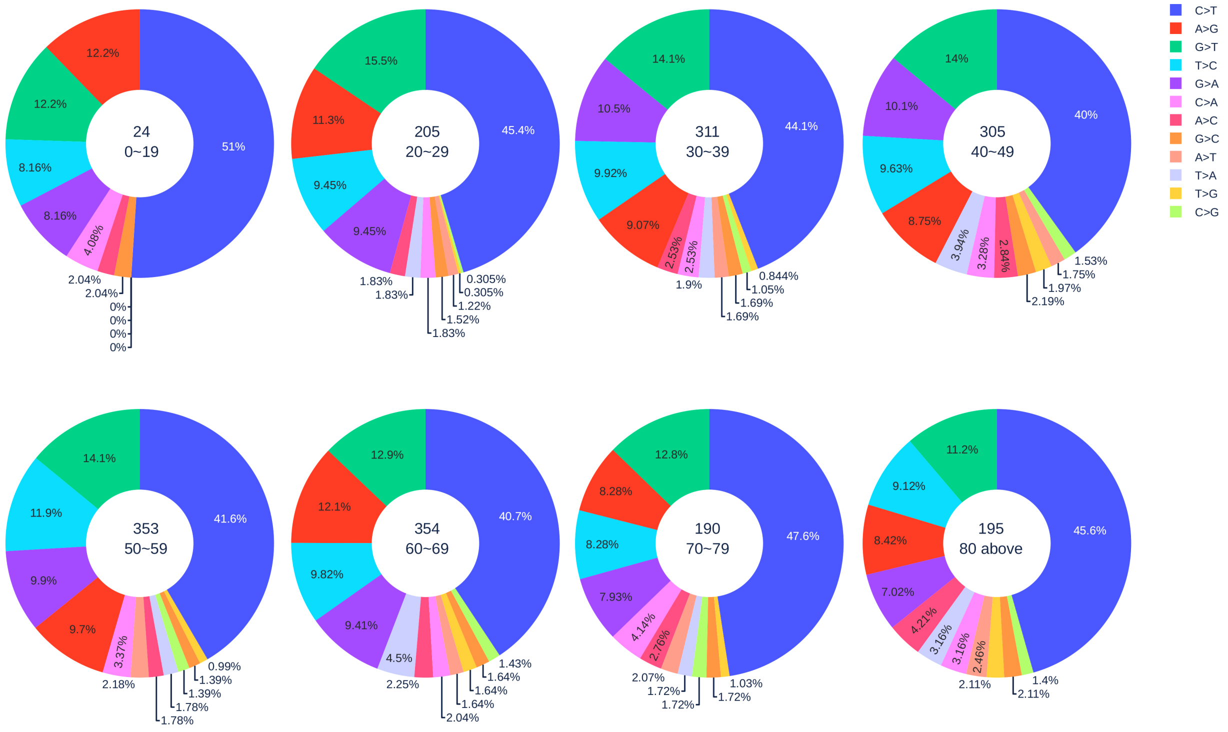

Figure S11: The distribution of 12 SNP types among unique mutations in the SARS-CoV-2 genome isolates from the United States. The text inside each circle represents for the total number of records in different age groups.

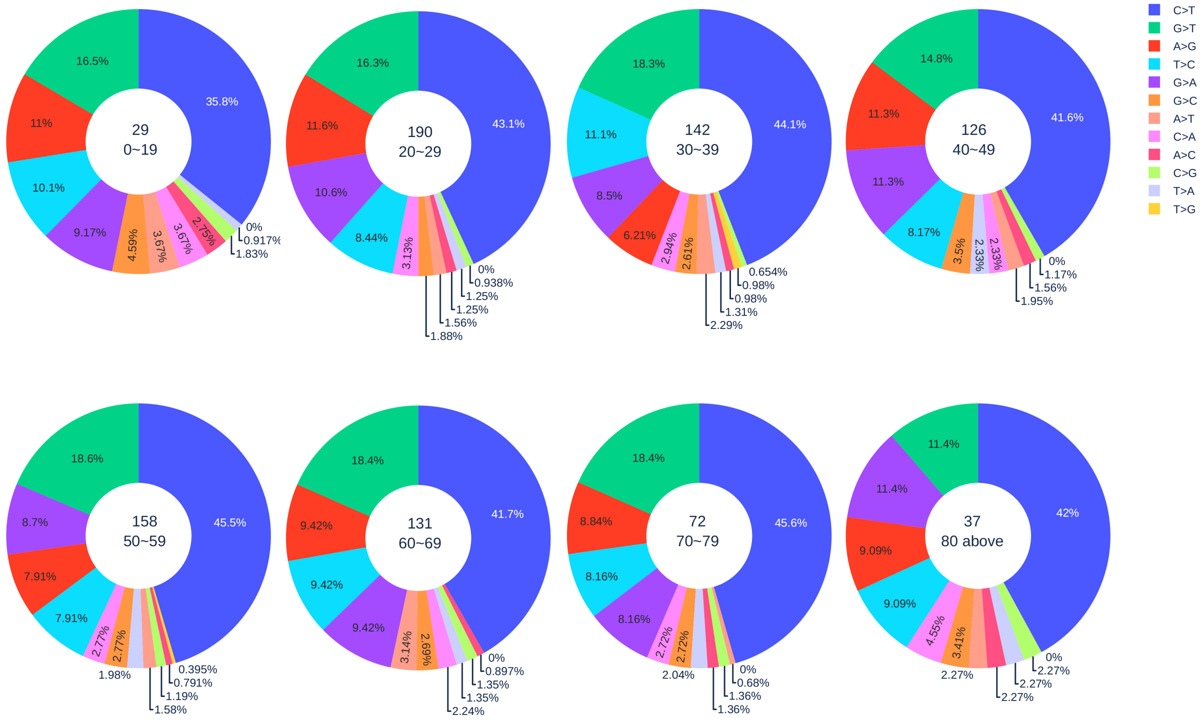

Figure S12: The distribution of 12 SNP types among unique mutations in the SARS-CoV-2 genome isolates from Australia. The text inside each circle represents for the total number of records in different age groups.

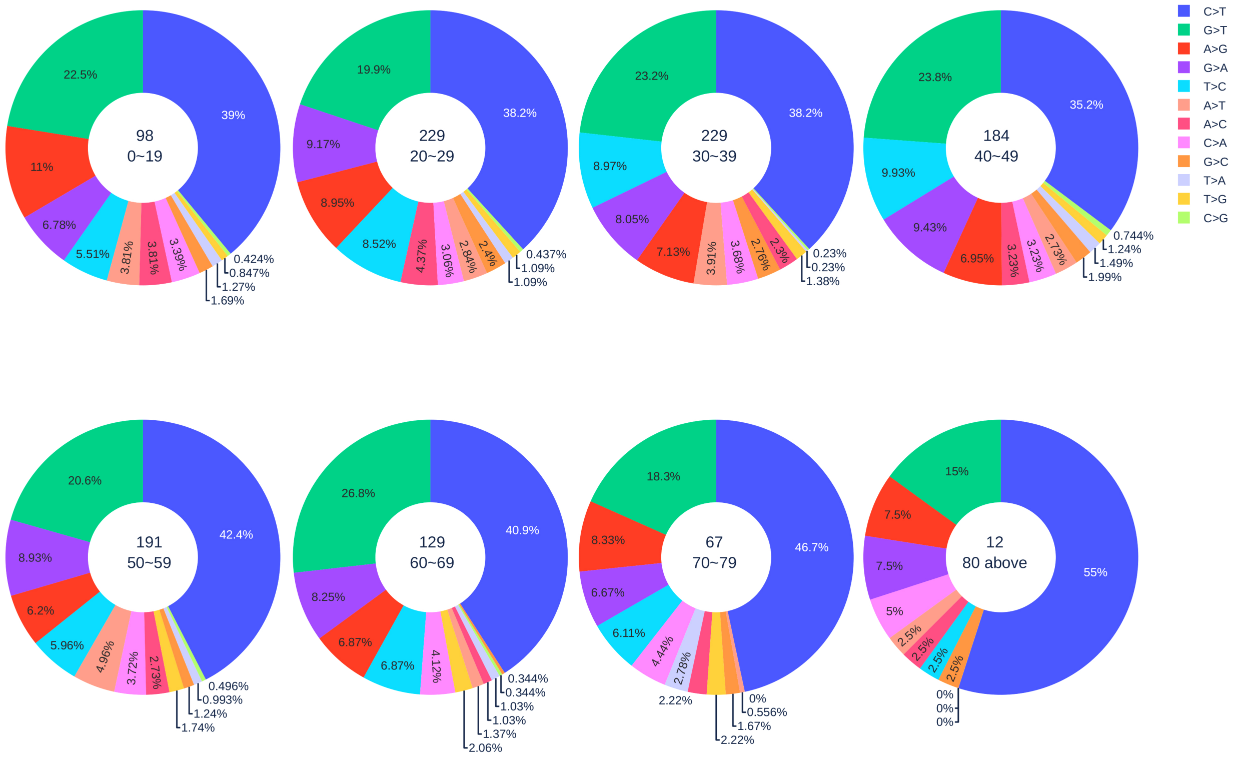

Figure S13: The distribution of 12 SNP types among unique mutations in the SARS-CoV-2 genome isolates from India. The text inside each circle represents for the total number of records in different age groups.

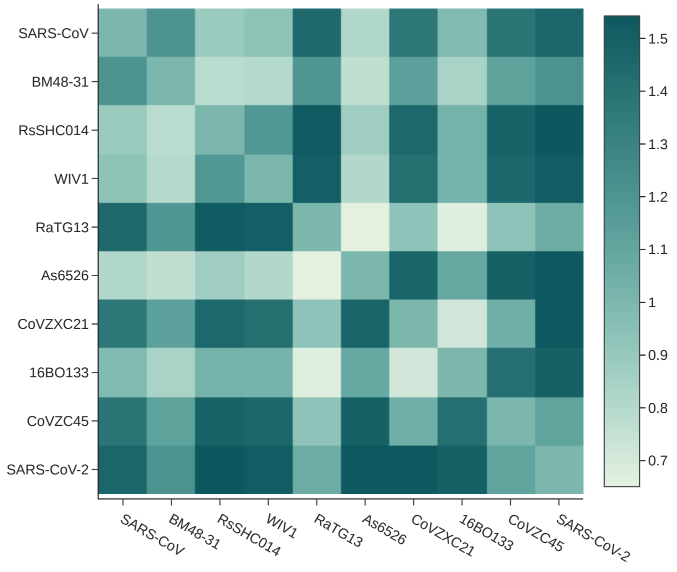

Figure S14: The heatmap of the C>T over T>C ratio between each pair of 10 different coronaviruses: SARS-CoV (2003), Bat-SL-BM48-31 (2008), Bat-SL-RsSHC014 (2011), Bat-SL-WIV1 (2012), Bat-RaTG13(2013), Bat-SL-As6526 (2014), Bat-SL-CoVZXC21 (2015), Bat-16BO133 (2016), Bat-SL-CoVZC45 (2017), SARS-CoV-2 (2019). For example, the colored square located in the SARS-CoV row and SARS-CoV-2 column represents the C>T over T>C ratio of the SARS-CoV-2 genome with the SARS-CoV genome as a reference.

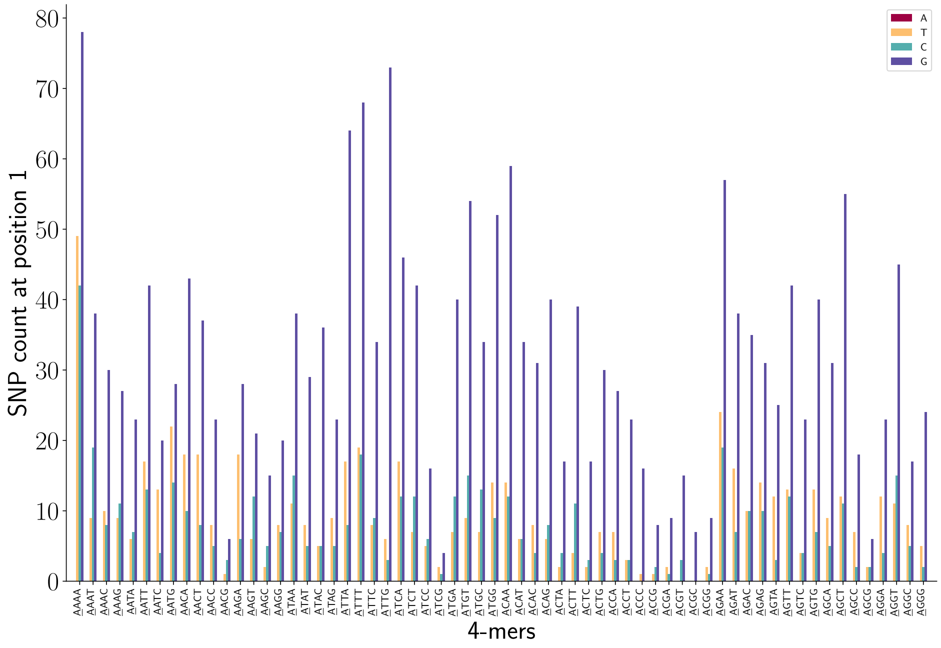

Figure S15: SNP frequencies at the first position of 4-mer motifs. Here, A is at the first position of 4-mer motifs.

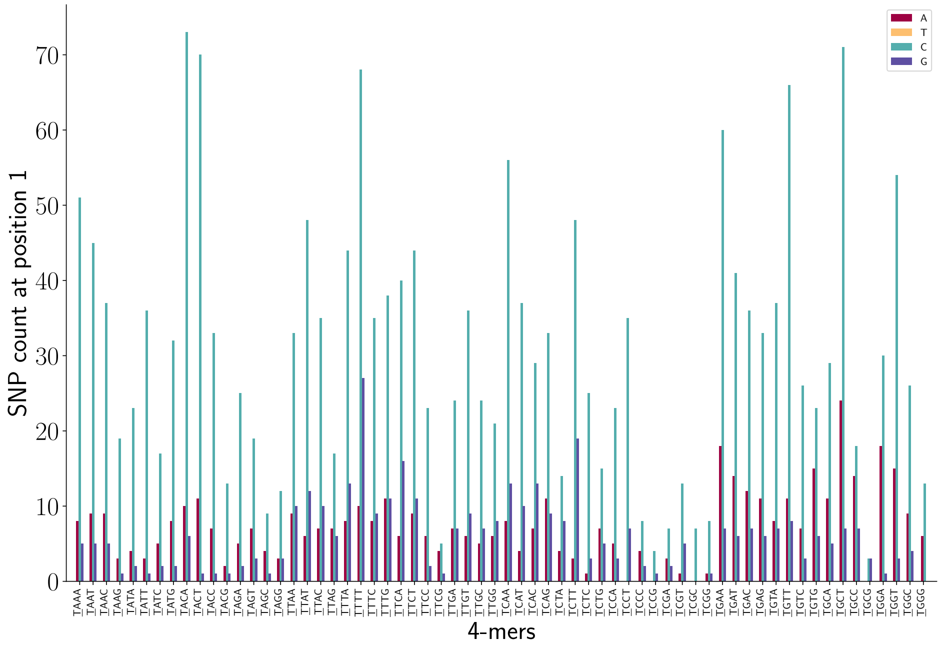

Figure S16: SNP frequencies at the first position of 4-mer motifs. Here, T is at the first position of 4-mer motifs.

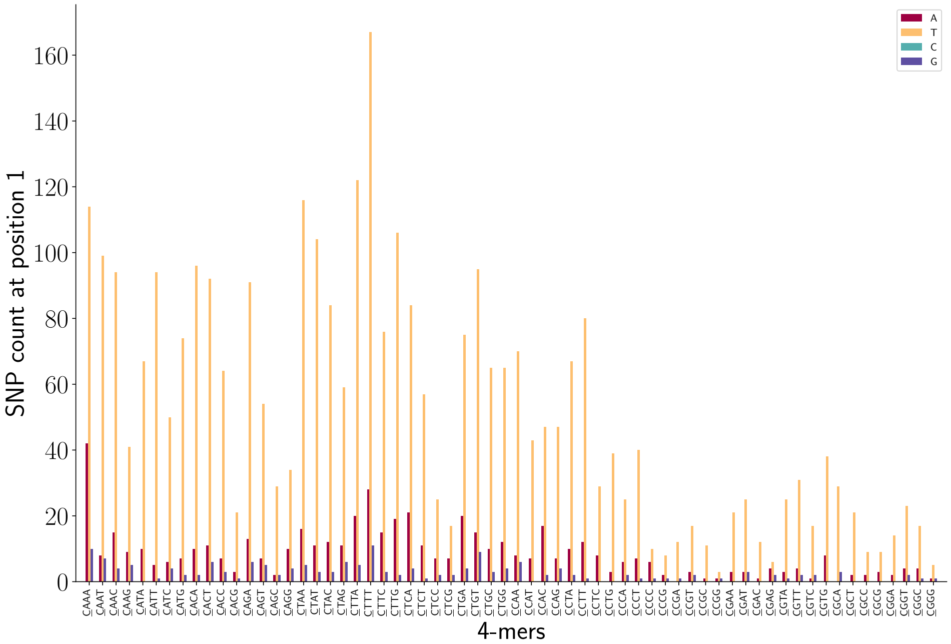

Figure S17: SNP frequencies at the first position of 4-mer motifs. Here, C is at the first position of 4-mer motifs.

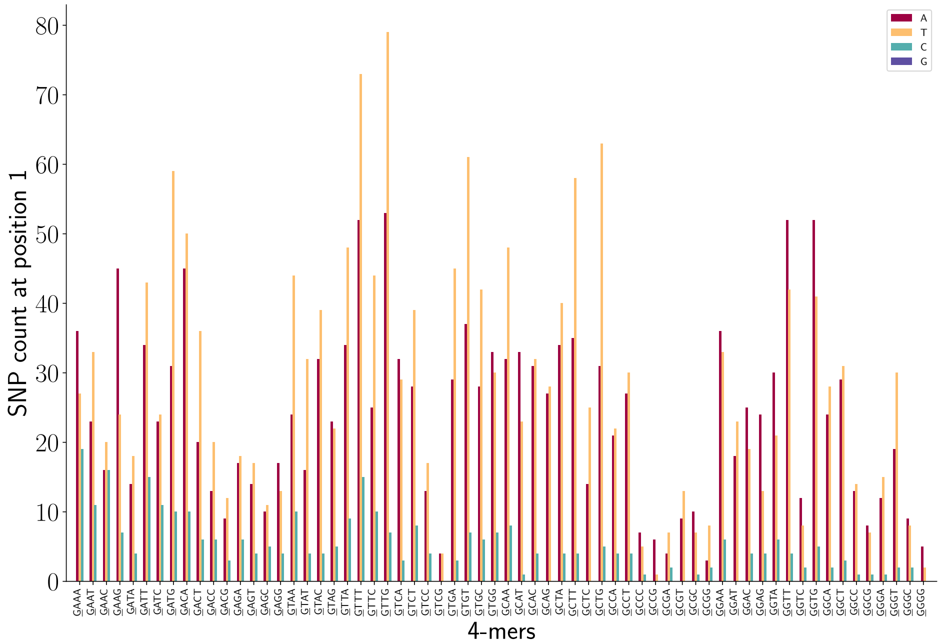

Figure S18: SNP frequencies at the first position of 4-mer motifs. Here, G is at the first position of 4-mer motifs.

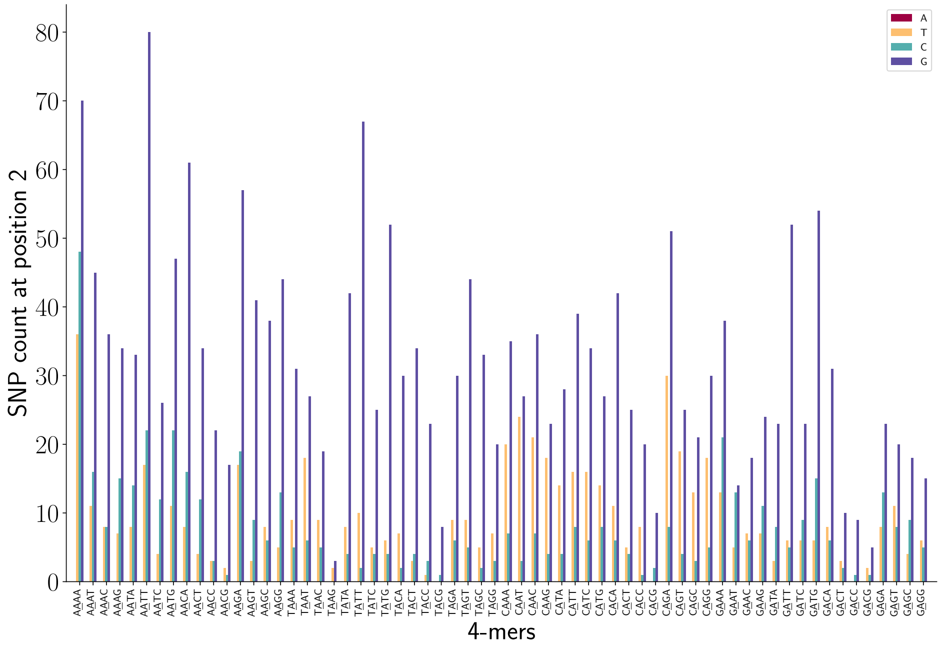

Figure S19: SNP frequencies at the second position of 4-mer motifs. Here, A is at the second position of 4-mer motifs.

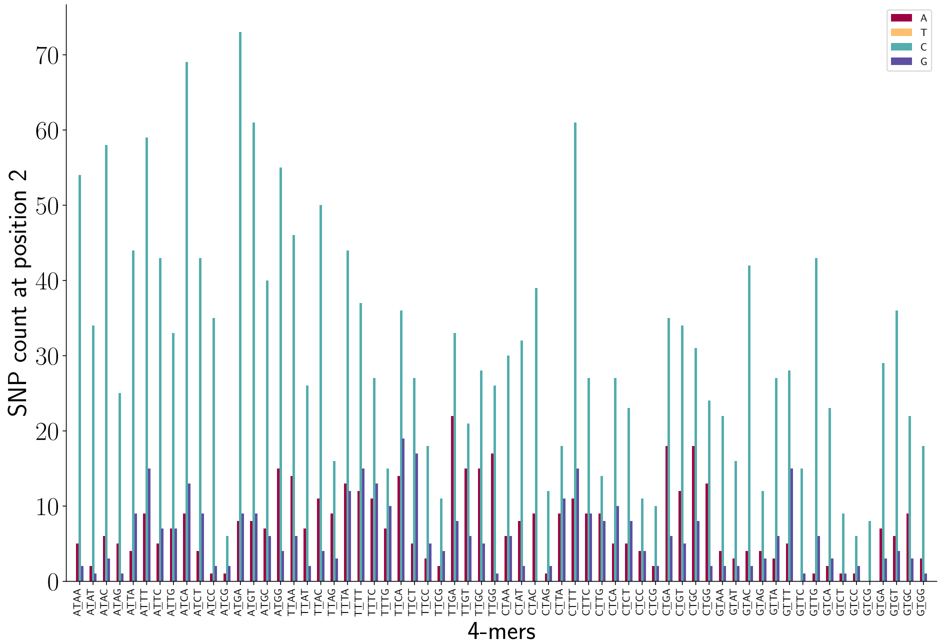

Figure S20: SNP frequencies at the second position of 4-mer motifs. Here, T is at the second position of 4-mer motifs.

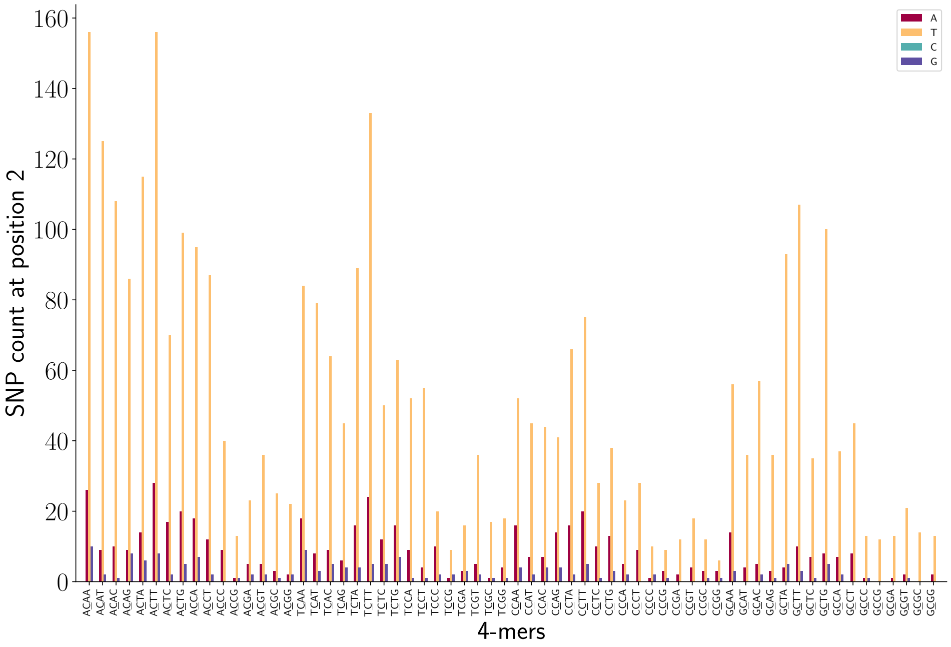

Figure S21: SNP frequencies at the second position of 4-mer motifs. Here, C is at the second position of 4-mer motifs.

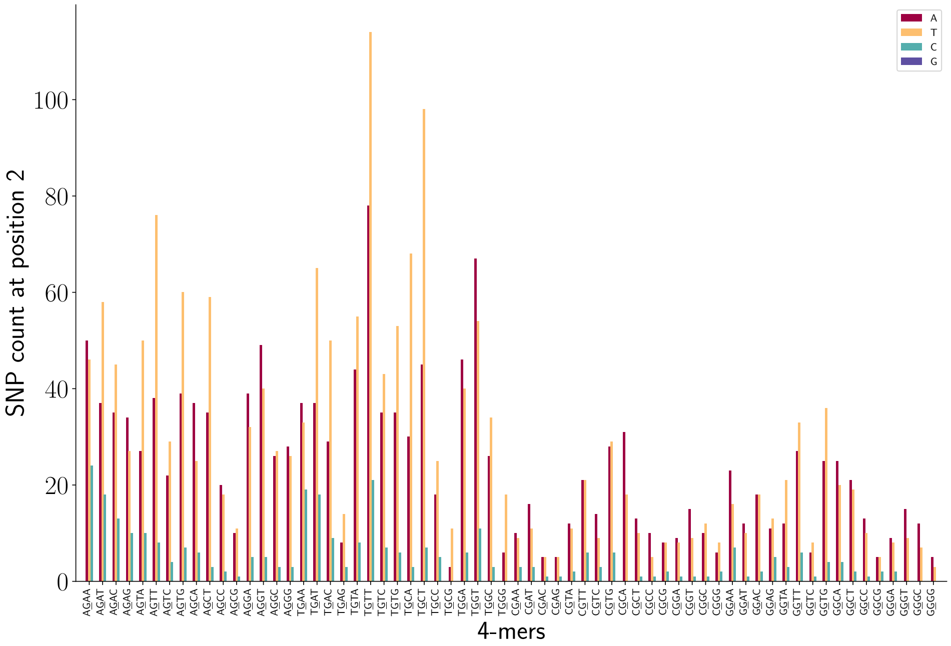

Figure S22: SNP frequencies at the second position of 4-mer motifs. Here, G is at the second position of 4-mer motifs.

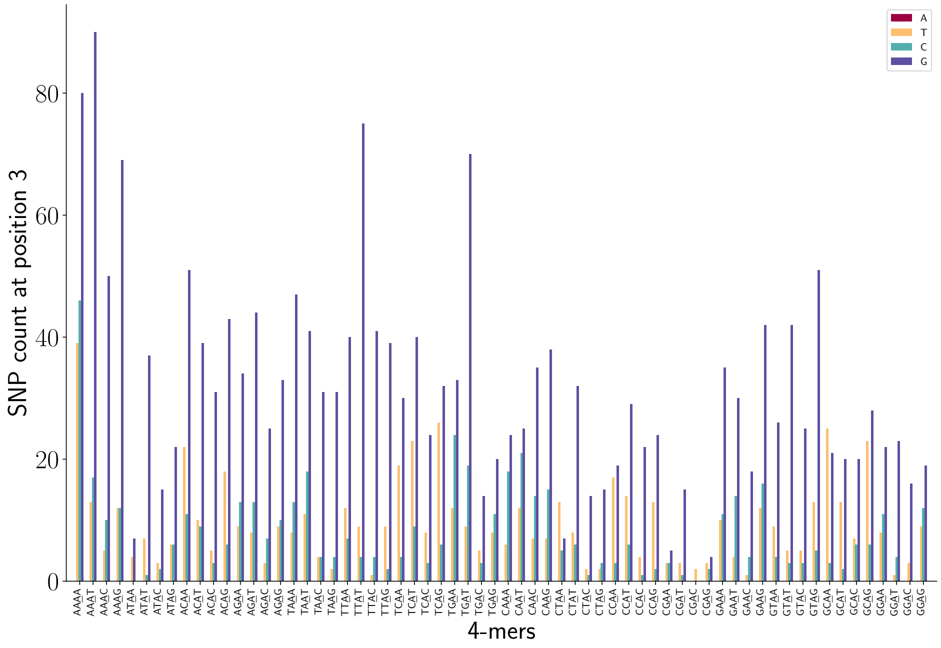

Figure S23: SNP frequencies at the third position of 4-mer motifs. Here, A is at the third position of 4-mer motifs.

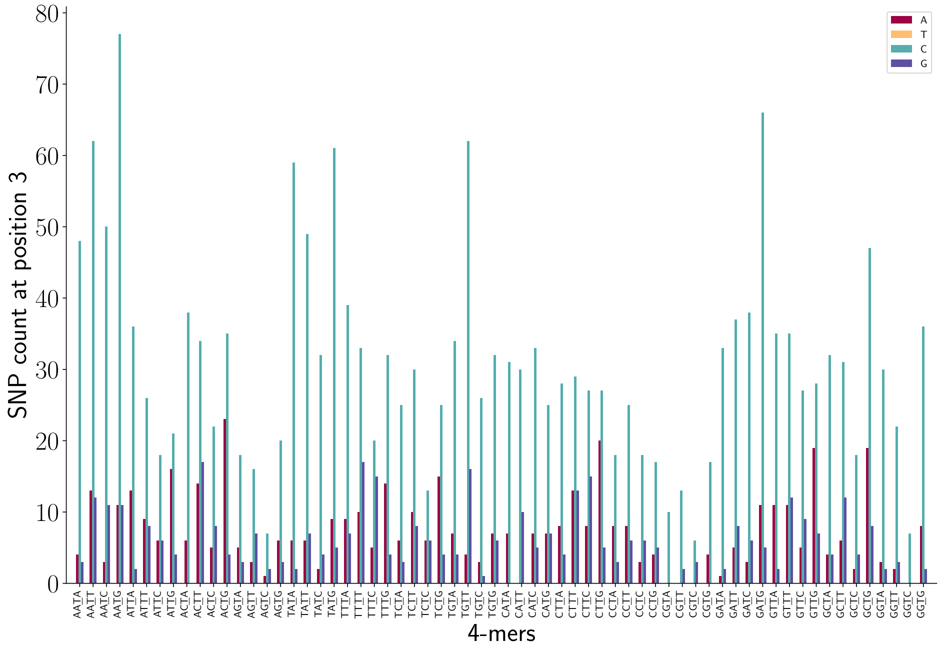

Figure S24: SNP frequencies at the third position of 4-mer motifs. Here, T is at the third position of 4-mer motifs.

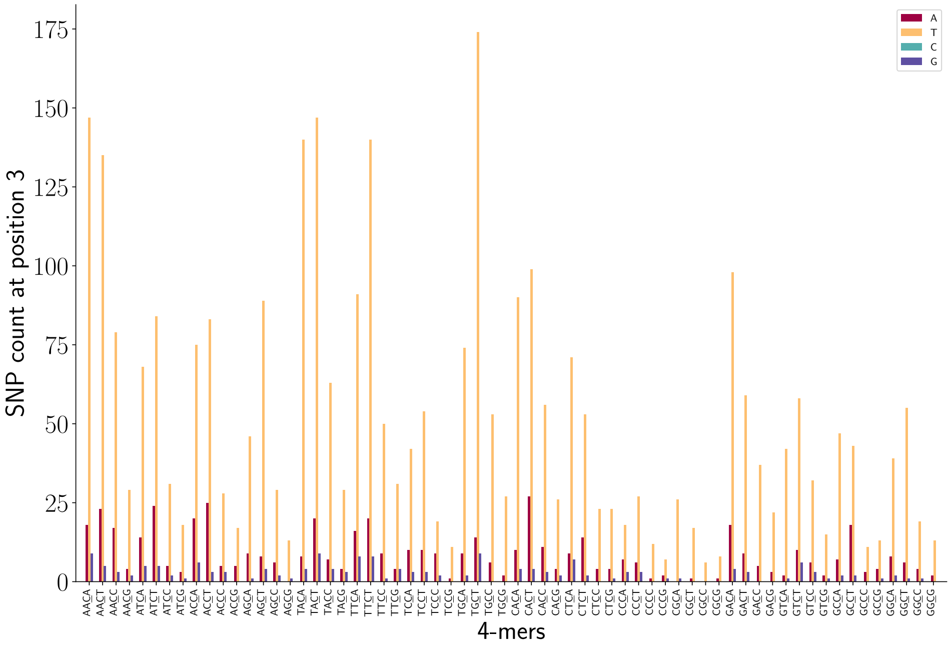

Figure S25: SNP frequencies at the third position of 4-mer motifs. Here, C is at the third position of 4-mer motifs.

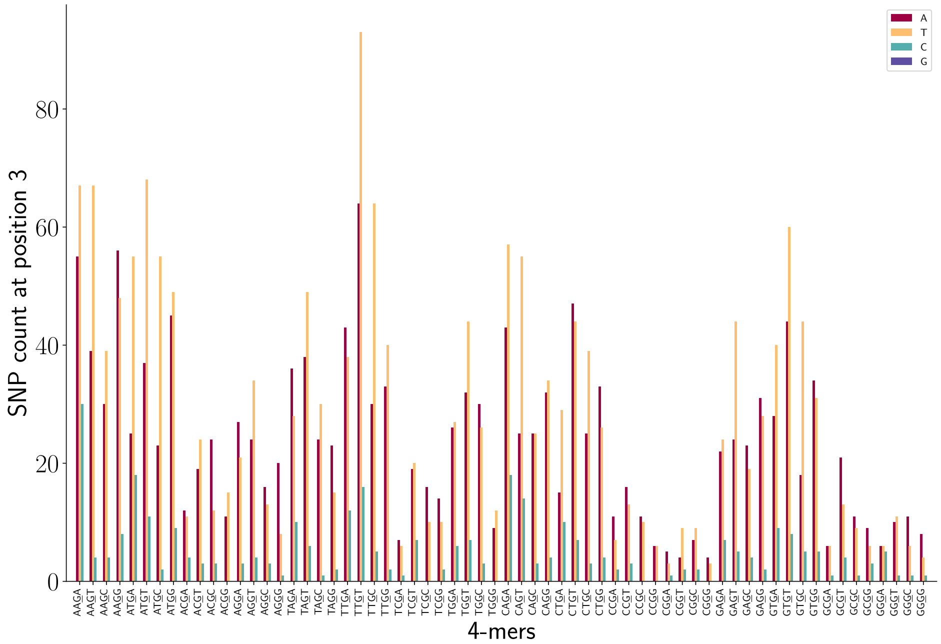

Figure S26: SNP frequencies at the third position of 4-mer motifs. Here, G is at the third position of 4-mer motifs.

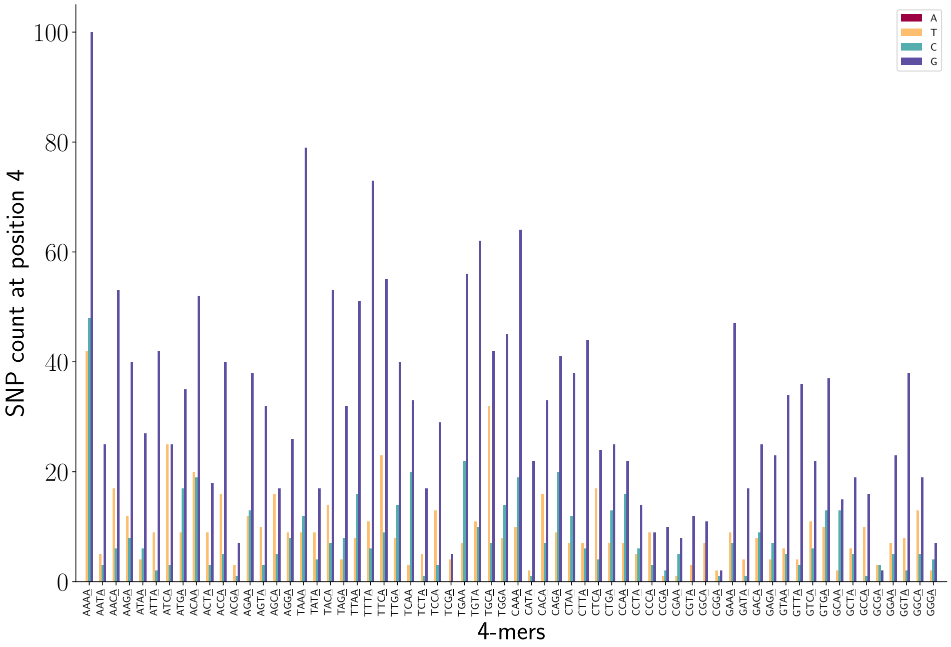

Figure S27: SNP frequencies at the fourth position of 4-mer motifs. Here, A is at the fourth position of 4-mer motifs.

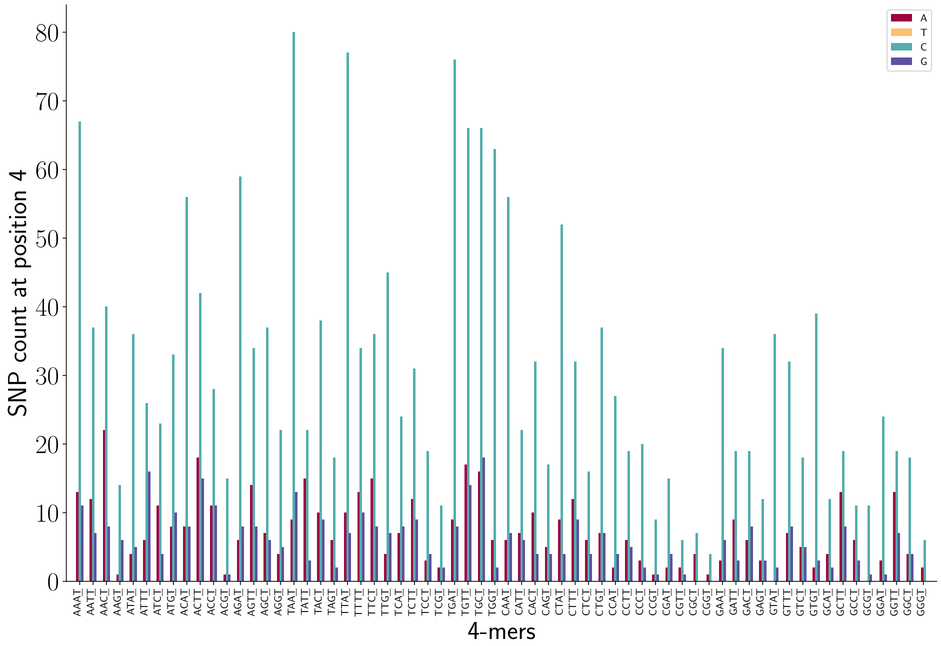

Figure S28: SNP frequencies at the fourth position of 4-mer motifs. Here, T is at the fourth position of 4-mer motifs.

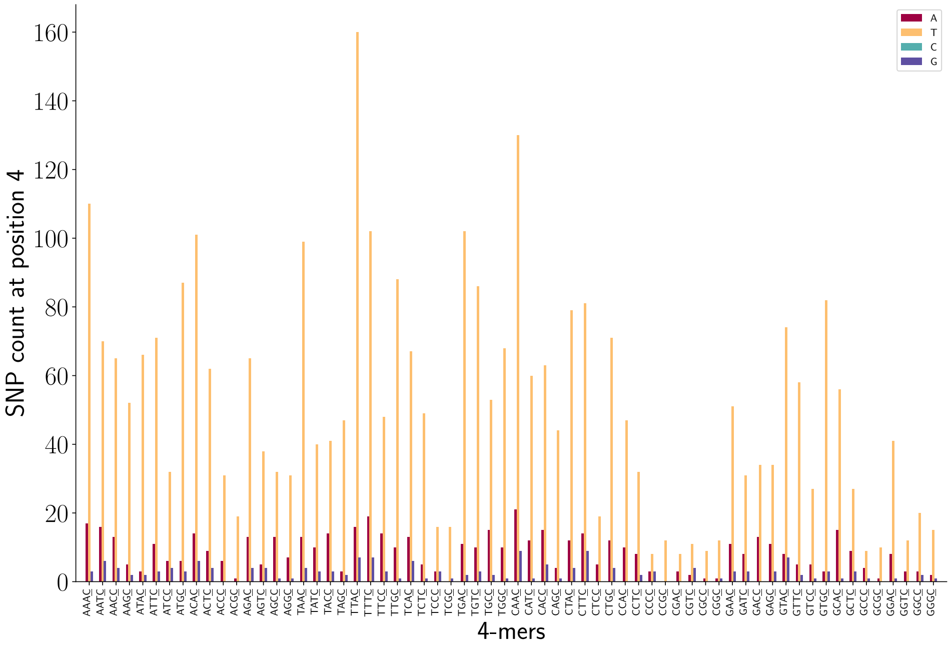

Figure S29: SNP frequencies at the fourth position of 4-mer motifs. Here, C is at the fourth position of 4-mer motifs.

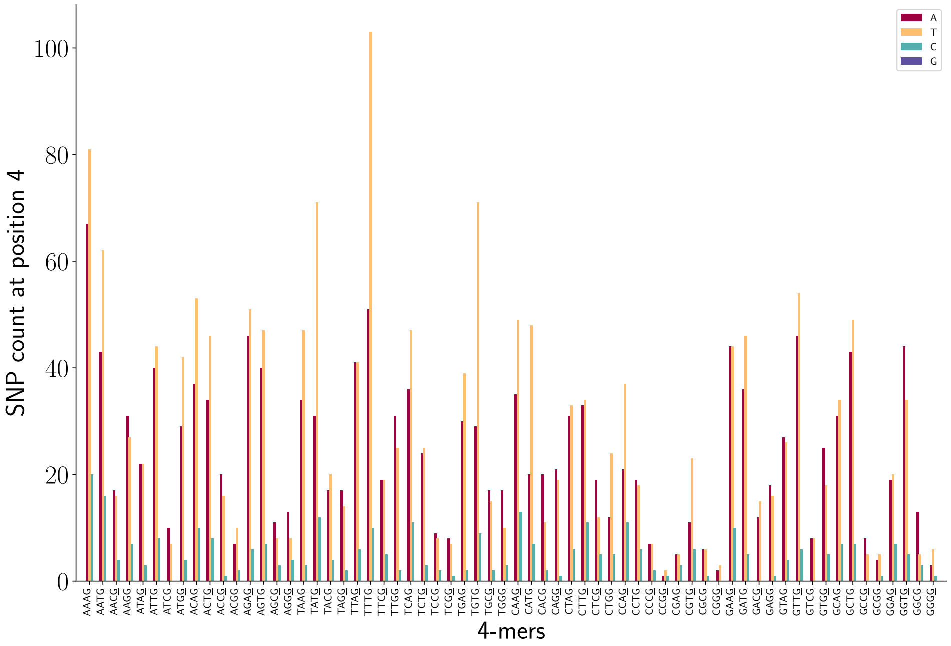

Figure S30: SNP frequencies at the fourth position of 4-mer motifs. Here, G is at the fourth position of 4-mer motifs.

S2 Supplementary Tables

Total 8 spreadsheets are merged in the Supporting_Tables.xlsx.

Table S1: Acknowledgement table provided by GISAID in Jan 2020.

Table S2: Acknowledgement table provided by GISAID in Feb 2020.

Table S3: Acknowledgement table provided by GISAID in March 2020.

Table S4: Acknowledgement table provided by GISAID in April 2020.

Table S5: Acknowledgement table provided by GISAID in May 2020.

Table S6: Acknowledgement table provided by GISAID in June 2020.

Table S7: Acknowledgement table provided by GISAID in July 2020.

Table S8: S8_GISAID_IDs_07312020: The GISAID IDs. (Up to July 31, 2020).

References

[1] Nelson Lee, David Hui, Alan Wu, Paul Chan, Peter Cameron, Gavin M Joynt, Anil Ahuja, Man Yee Yung, CB Leung, KF To, et al. A major outbreak of severe acute respiratory syndrome in Hong Kong. *New England Journal of Medicine*, 348(20):1986–1994, 2003.

[2] Jan Felix Drexler, Florian Gloza-Rausch, Jörg Glende, Victor Max Corman, Doreen Muth, Matthias Goettsche, Antje Seebens, Matthias Niedrig, Susanne Pfefferle, Stoian Yordanov, et al. Genomic characterization of severe acute respiratory syndrome-related coronavirus in European bats and classification of coronaviruses based on partial RNA-dependent RNA polymerase gene sequences. *Journal of virology*, 84(21):11336–11349, 2010.

[3] Xing-Yi Ge, Jia-Lu Li, Xing-Lou Yang, Aleksei A Chmura, Guangjian Zhu, Jonathan H Epstein, Jonna K Mazet, Ben Hu, Wei Zhang, Cheng Peng, et al. Isolation and characterization of a bat SARS-like coronavirus that uses the ACE2 receptor. *Nature*, 503(7477):535–538, 2013.

[4] Peng Zhou, Xing-Lou Yang, Xian-Guang Wang, Ben Hu, Lei Zhang, Wei Zhang, Hao-Rui Si, Yan Zhu, Bei Li, Chao-Lin Huang, et al. A pneumonia outbreak associated with a new coronavirus of probable bat origin. *Nature*, 579(7798):270–273, 2020.

[5] Ben Hu, Lei-Ping Zeng, Xing-Lou Yang, Xing-Yi Ge, Wei Zhang, Bei Li, Jia-Zheng Xie, Xu-Rui Shen, Yun-Zhi Zhang, Ning Wang, et al. Discovery of a rich gene pool of bat SARS-related coronaviruses provides new insights into the origin of SARS coronavirus. *PLoS pathogens*, 13(11):e1006698, 2017.

[6] Dan Hu, Changqiang Zhu, Lele Ai, Ting He, Yi Wang, Fuqiang Ye, Lu Yang, Chenxi Ding, Xuhui Zhu, Ruicheng Lv, et al. Genomic characterization and infectivity of a novel SARS-like coronavirus in Chinese bats. *Emerging Microbes & Infections*, 7(1):1–10, 2018.

[7] Fan Wu, Su Zhao, Bin Yu, Yan-Mei Chen, Wen Wang, Zhi-Gang Song, Yi Hu, Zhao-Wu Tao, Jun-Hua Tian, Yuan-Yuan Pei, et al. A new coronavirus associated with human respiratory disease in China. *Nature*, 579(7798):265–269, 2020.
